# A description of teaching methods using an on-site instructor versus a distant site instructor to train laryngoscopy to medical students in Hanoi, Vietnam, from Omaha, Nebraska, by video communication

**DOI:** 10.1186/s12245-015-0085-0

**Published:** 2015-12-01

**Authors:** Wesley G. Zeger, Chad E. Branecki, Thang T. Nguyen, Todd Hall, Ben Boedeker, David Boedeker, Michael C. Wadman

**Affiliations:** University of Nebraska Medical Center, Omaha, NE USA; Landstuhl Regional Medical Center, Landstuhl, DE Germany; Columbia University Mailman School of Public Health, New York, NY USA

## Abstract

This study demonstrated a method to train medical students at Hanoi Medical School in airway management from Omaha, Nebraska, using tele-mentoring techniques. Correct placement of the endotracheal tube was documented by tele-broncoscopy following intubation. This technology may increase medical training capabilities in remote or developing areas of the world. Medical care delivery could be performed using this technology by tele-mentoring a lesser trained medical provider at a distant site enabling them to accomplish complex medical tasks.

## Introduction

Currently, most procedural skills instruction for healthcare providers in Vietnam, as well as in most other developing countries, is provided by on-site personnel. The logistical challenges of placing skilled instructors at these distant sites limit the global dissemination of the latest best practices in patient care, and strategies are needed to expand the impact of high-quality instructors, advances in medical knowledge, and new technologies.

Training in airway management is a critical skill in medical education. With improved internet resources, virtual training from a medical center to remote sites may provide an efficient and cost effective way to perform essential skills training.

Previous studies describe student education in video intubation using on-site trainers and documented proper endotracheal tube placement by tele-broncoscopy, with a remote teacher providing real time guidance to the learner as the procedure is performed [1–3]. Confirmation of successful completion of the skill is necessary to ensure effective instruction. This study describes both instructions in laryngoscopy using a video laryngoscope to allow for off-site tele-mentoring and off-site confirmation of correct endotracheal tube placement by tele-broncoscopy. The methodology of this study would allow the entire laryngoscopy training to be performed virtually.

## Methods

**Fig. 1:**
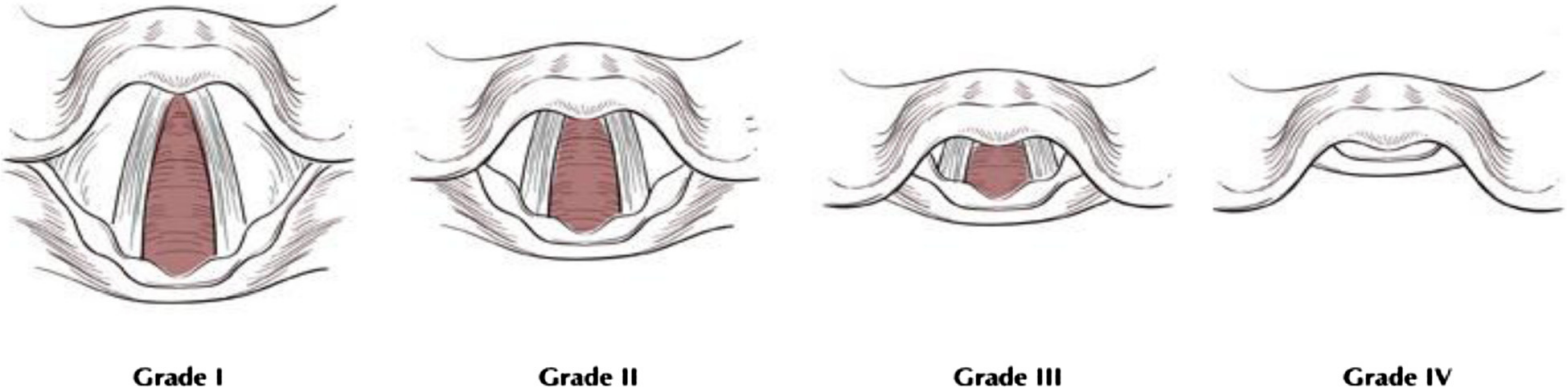
Cormack/Lehane views of the glottic opening as seen during laryngoscopy

After IRB approval, an audiovisual link using Vidyo, a HIPPA secure conferencing program (Vidyo, Inc., Hackensack, NJ) link was established between the Center for Advanced Technology and Telemedicine (CATT), University of Nebraska Medical Center (UNMC) in Omaha, Nebraska, and Hanoi Medical University (HMU) in Hanoi, Vietnam. Medical students in Hanoi were instructed via tele-mentoring by an instructor at CATT in Omaha, Nebraska, in how to perform an intubation. After receiving intubation instruction, students performed one intubation on an airway mannequin. The instruction session consisted of visual demonstration of the critical anatomic structures only, and the instructor refrained from any physical guidance with the students’ intended to modify their laryngoscope grasp, lifting force trajectory, or intubating positions. The intubation was done using a Karl Storz video laryngoscope with a #3 Macintosh blade. Students were timed in how long it took to perform the intubation. The Cormack/Lehane (C/L) view (Fig. 1) of the glottic opening observed by the student was recorded [4]. For the standard, on-site mentoring group in January 2015, eight medical student subjects performed video intubations after having been instructed by an on-site instructor. For the tele-mentoring group in March 2015, eight medical student subjects performed video intubations after instruction by an off-site instructor at CATT in Omaha, Nebraska, linked to HMU by videoconference. After completing the intubation, a bronchoscope, (linked to the Vidyo program by a Karl Storz C CAM and C HUB) was placed in the endotracheal lumen and advanced to the tip of the tube for confirmation of correct placement by the distant instructor at CATT. Proper endotracheal tube placement was confirmed by visualization of the tracheal rings, carina, or esophagus, depending on the level of the tip of the endotracheal tube (Fig. 1). Additional confirmation of correct tube placement was performed by the on-site instructor in Hanoi. Success or nonsuccess for each intubation was recorded. Teaching was in English, and the Vietnamese medical students spoke English as a second language. In terms of the statistical methods, researchers used averages to compare the two groups of data. This was deemed the best method for statistical comparison since it provides a holistic view of all participants.

## Results

For the January 2015 standard on-site mentoring subjects, eight HMU medical students performed video intubations after having been instructed by an on-site instructor. As shown in Table 1, they had 75 % success rate as documented by tracheal rings observation by tele-broncoscopy. The average intubation time was 39.3 s (Table 1).

**Table 1:** Direct mentoring trial: airway training performed by on-site instructor on-site at HMU

	C/L view grade	Intubation time	Result	Confirmation method
Subject 1	1	34 s	+	Tracheal rings
Subject 2	1	42 s	+	Tracheal rings
Subject 3	1	24 s	+	Tracheal rings
Subject 4	NA	23 s	–	NA
Subject 5	1	16 s	+	Tracheal rings
Subject 6	1	43 s	+	Tracheal rings
Subject 7	3	108 s	–	NA
Subject 8	2	25 s	+	Tracheal rings
Total	5, grade 1; 1, grade 2; 1, grade 3; 0, grade 4; 1 NA	Average, 39.3 s	75 % success, 25 % fail	6-tracheal rings, 2-NA

For the March 2015 tele-mentoring subjects, 11 HMU medical students performed video intubations after instruction by an off-site instructor at CATT in Omaha, Nebraska, linked to HMU by videoconference. Their success rate was 81.2 %, and average intubation time was 290 s (Table 2).

**Table 2:** Tele-mentoring trial: airway training performed by distant instructor at CATT/UNMC in Omaha, Nebraska, connecting to student at HMU by Vidyo link

	C/L view grade	Intubation time	Result	Confirmation method
Subject 1	1	97 s	+	Carina
Subject 2	NA	242 s	−	NA
Subject 3	3	231 s	−	NA
Subject 4	2	417 s	+	Carina
Subject 5	1	335 s	+	Carina
Subject 6	2	293 s	+	Carina
Subject 7	3	354 s	+	Carina
Subject 8	2	66 s	+	Carina
Subject 9	3	93 s	+	Carina
Subject 10	2	180 s	+	Carina
Subject 11	2	885 s	+	Carina
Total	3, grade 1; 5, grade 2; 3, grade 3; 0, grade 4; 1 NA	Average, 290.2 s	9 (81.8 %) success, 2 (18.1 %) fail	9-carina, 2-NA

## Discussion

One significant limitation of the study was the variable experience in airway management between the subject groups. Following completion of the experiment, the on-site instructors in Hanoi were informed that the second subject group in March had limited experience with intubation or laryngoscopy, compared to some significant laryngoscopy and intubating experience for the students in the first subject group participating in January.

As this is a preliminary report, the student numbers are too small to derive any statistical comparison between on-site versus distant site instruction. Review of the data shows that off-site, distant instruction appears to have resulted in longer times for intubation. As more students are trained by this project, using both on-site and distant site instruction, greater statistical comparison of the methods will be possible. However, this study demonstrates the feasibility of tele-mentoring for even the most novice learner in laryngoscopy.

## Conclusion

This project demonstrates airway management instruction for medical students using on-site instructors versus tele-mentoring by distant site instructors. Of note, on-site instruction of the January subject group was only visual demonstration of the critical anatomy structures, and the instructor had no physical contact with the students to modify the subject’s intubating position or posture. The lack of any hands-on modification of the learners’ grasp, positioning, and posture during the procedure is important in comparing on-site mentoring to tele-mentoring from the distant site. Future studies may define a role for applied virtual reality in guidance of these psychomotor actions.
